# Harnessing the potential of integrated systematics for conservation of taxonomically complex, megadiverse plant groups

**DOI:** 10.1111/cobi.13289

**Published:** 2019-02-19

**Authors:** Eimear M. Nic Lughadha, Vanessa Graziele Staggemeier, Thais N. C. Vasconcelos, Barnaby E. Walker, Cátia Canteiro, Eve J. Lucas

**Affiliations:** ^1^ Royal Botanic Gardens, Kew TW9 3AE Richmond Surrey U.K.; ^2^ Universidade Estadual Paulista, Instituto de Biociências, Departamento de Botânica Laboratório de Fenologia Avenida 24A 1515, CEP 13506–900 Rio Claro São Paulo Brazil; ^3^ Departamento de Ecologia, Centro de Biociências Universidade Federal do Rio Grande do Norte CEP 59072–970 Natal Rio Grande do Norte Brazil; ^4^ Laboratório de Sistemática Vegetal Departamento de Botânica, Universidade de São Paulo, São Paulo SP 05508–090 Brazil

**Keywords:** extent of occurrence, extinction risk, georeferenced, herbarium, IUCN Red List, misidentification, monography, taxonomic remodeling, extensión de la distribución, georeferenciación, herbario, identificación errónea, Lista Roja UICN, monografía, remodelación taxonómica

## Abstract

The value of natural history collections for conservation science research is increasingly recognized, despite their well‐documented limitations in terms of taxonomic, geographic, and temporal coverage. Specimen‐based analyses are particularly important for tropical plant groups for which field observations are scarce and potentially unreliable due to high levels of diversity‐amplifying identification challenges. Specimen databases curated by specialists are rich sources of authoritatively identified, georeferenced occurrence data, and such data are urgently needed for large genera. We compared entries in a monographic database for the large Neotropical genus *Myrcia* in 2007 and 2017. We classified and quantified differences in specimen records over this decade and determined the potential impact of these changes on conservation assessments. We distinguished misidentifications from changes due to taxonomic remodeling and considered the effects of adding specimens and georeferences. We calculated the potential impact of each change on estimates of extent of occurrence (EOO), the most frequently used metric in extinction‐risk assessments of tropical plants. We examined whether particular specimen changes were associated with species for which changes in EOO over the decade were large enough to change their conservation category. Corrections to specimens previously misidentified or lacking georeferences were overrepresented in such species, whereas changes associated with taxonomic remodeling (lumping and splitting) were underrepresented. Among species present in both years, transitions to less threatened status outnumbered those to more threatened (8% vs 3%, respectively). Species previously deemed data deficient transitioned to threatened status more often than to not threatened (10% vs 7%, respectively). Conservation scientists risk reaching unreliable conclusions if they use specimen databases that are not actively curated to reflect changing knowledge.

## Abstract 2

*Article impact statement*: Digitized natural history collections must be updated as taxonomic knowledge grows so as to maximize their utility for conservation science.

## Abstract (es)

Empleo del Potencial de la Sistemática Integrada para la Conservación de Grupos Botánicos Complejos y Megadiversos

**Resumen**

Cada vez se reconoce más el valor que tienen las colecciones de historia natural para la investigación dentro de la ciencia de la conservación, a pesar de las limitaciones en la documentación adecuada de la cobertura taxonómica, geográfica y temporal. Los análisis con base en especímenes son de particular importancia en los grupos de plantas tropicales, para los cuales las observaciones en el campo son escasas y potencialmente de poca confianza debido la gran cantidad de retos de identificación que amplifican la diversidad. Las bases de datos de especímenes que son curadas por especialistas son fuentes abundantes de datos identificados autoritativamente y de distribución georeferenciada, por lo que es urgente la necesidad de dichos datos para géneros extensos. Comparamos las entradas en una base de datos monográfica para el género neotropical extenso *Myrcia* en 2007 y en 2017. Clasificamos y cuantificamos las diferencias en los registros de especímenes durante esta década y determinamos el impacto potencial de estos cambios sobre las evaluaciones de conservación. Separamos las identificaciones erróneas de los cambios causados por la remodelación taxonómica y consideramos los efectos de la adición de especímenes y de georeferencias. Calculamos el impacto potencial de cada cambio sobre las estimaciones de la extensión de la distribución (EOO, en inglés), la medida que se utiliza con mayor frecuencia en las valoraciones del riesgo de extinción de las plantas tropicales. Examinamos si los cambios en un espécimen particular estuvieron asociados con especies para las cuales los cambios en la EOO durante la década fueron lo suficientemente amplios para cambiarles la categoría de conservación. Las correcciones hechas a especímenes previamente mal identificados o carentes de georeferencias estuvieron sobre‐representadas en dichas especies, mientras que los cambios asociados con la remodelación taxonómica (agrupamiento y separación) estuvieron sub‐representados. Entre las especies presentes en ambos años, las transiciones hacia un estado de conservación con menor amenaza superaron en cantidad a aquellas hacia un estado de mayor amenaza (8% vs 3%, respectivamente). Las especies que con anterioridad se clasificaban como deficientes de datos tuvieron más transiciones hacia el estado de amenazadas que hacia el estado de no amenazadas (10% vs 7%, respectivamente). Los científicos de la conservación corren el riesgo de llegar a conclusiones poco confiables si utilizan bases de datos de especímenes que no sean actualizadas para reflejar el conocimiento cambiante.

## Introduction

The utility of natural history collections (NHCs) for conservation research has long been recognized (Ponder et al. 2001; Gaubert et al. 2006). Although some authors have outlined the limitations of data derived from NHCs in quantitative (Meyer et al. 2016) and qualitative (Graham et al. 2004; Daru et al. 2018) terms, emphasizing gaps, biases, and uncertainties in taxonomic, geographic, and temporal dimensions of such data, the irreplaceable value of NHCs to research on the future of life on Earth is increasingly recognized (Lavoie 2013; Nualart et al. 2017). Calls for accelerated digital access to NHCs are frequently justified by their relevance to conservation (e.g., Greve et al. 2016).

Although systematic studies have driven the development of most NHCs and the databases in which NHCs are recorded, a growing proportion of collections‐based research focuses on conservation. Such studies range from status recognition of threatened, rare, or declining species (Rivers et al. 2011), conservation prioritization (Murray‐Smith et al. 2009), and the impact of climate change on plant phenology (Jones & Daehler 2018), the latter representing a particularly rapidly growing research area in plants (Lavoie 2013) that has many potential applications in conservation (Morellato et al. 2016). Compilation of phenological calendars is also a key to plan collection of mature seeds for ex situ conservation, and an understanding of interactions between fruiting phenology, seed‐storage behavior, dormancy, and germination informs their effective preservation. For all these examples, accurate identification of the specimens from which the data are collected is crucial to the integrity of the resulting analysis.

Specimen‐based approaches offer particularly exciting prospects in the tropics, where field observations are scarce (Morellato et al. 2016). Furthermore, high plant diversity in tropical environments makes it difficult or impossible to detect identification errors in unvouchered field observations. Data from NHCs can be audited, corrected, enriched, or refuted in light of further study of the original voucher specimens on which they are based. In short, specimens provide the basis of reproducibility, an essential element of the scientific method but one that has eroded to the extent that many scientists now perceive a significant crisis of reproducibility.

Important though they are, NHCs are just part of the story. Accurate identifications require clear, well‐documented species circumscriptions and, in the case of large or complex groups, specialists familiar with diagnostic characters at all taxonomic ranks. Such expertise often lies with monographers preparing definitive treatments of all species in a genus or family. The resulting monographs and intensively curated specimen data sets on which they are founded arguably provide the highest quality resources possible on which to base conservation analyses (Landrum 2001). However, monographs can take years, or decades, to complete, especially for large genera that contribute so much to tropical plant ecosystems in both species numbers and diversity. In the light of current and projected global environmental change, few scientists would advocate awaiting publication of monographs including finalized taxonomic units before treating taxonomically complex groups in conservation analyses. However, premature analyses demonstrably based on poorly defined or unrecognizable units will likely not inform effective conservation action. Can scientists involved in monography and conservation assessment identify approaches that play to their strengths in finding the appropriate balance between acting now and waiting for more information? Just how much difference do improved species circumscriptions and identifications make to understand extinction risk in large complex groups?

We addressed these questions qualitatively and quantitatively for Neotropical Myrtaceae. With 6040 species (Govaerts et al. 2018), Myrtaceae is the seventh largest angiosperm family. Myrtaceae are critical elements of tropical ecosystems that provide multiple resources to pollinators and dispersers (Gressler et al. 2006), are a useful group for modeling total species diversity and dynamics in some tropical biomes (Murray‐Smith et al. 2009), and are a potential tool for ecosystem management strategy (Rigueira et al. 2013). Myrtaceae are common throughout much of the tropics and include megadiverse, taxonomically complex genera (such as, *Eugenia*, *Myrcia*, *Eucalyptus*, and *Syzygium*). These giants exhibit extreme floral trait homoplasy (Vasconcelos et al. 2015), which has made classification difficult for over 2 centuries (Lucas & Bünger 2015). *Myrcia*, here used in the sense of Lucas et al. (2018), including species formerly treated as *Calyptranthes, Gomidesia, Marlierea*, and *Mitranthes*, is the largest exclusively Neotropical genus in the family (c. 800 species). *Myrcia* is currently the focus of collaborative monographic effort and a linked initiative to evaluate the extinction risk of all known species by 2021. To evaluate how NHCs in association with thorough taxonomic revisions underpin accurate conservation assessments in a tropical group, we analyzed changes in databased *Myrcia* specimens before and after 10 years of intensive systematic input. We considered changes in species taxonomy, specimen georeferencing, conservation status, and diversity distribution.

## Methods

### *Myrcia* Specimen Database

We extracted the specimen data set used here from a database of 18,805 *Myrcia* specimens compiled and curated at the Royal Botanic Gardens, Kew. The database includes label data transcribed from all *Myrcia* specimens in the herbaria of Kew, New York Botanical Garden, National Herbarium of French Guiana, Jardim Botânico do Rio de Janeiro, and National Herbarium Nederland, Utrecht. Specimens from online herbaria are also included as are label data transcribed from specimens during herbarium visits or from publications. Only specialist‐verified specimens are included in the database, so over 100 rare or poorly collected species are not represented within it. Records were georeferenced to the nearest latitude–longitude minute based on online resources (http://www.fallingrain.com; http://middleware.alexandria.ucsb.edu). Taxonomy follows Govaerts et al. (2018), updated with the Flora do Brasil (JBRJ 2018) and complemented by published and unpublished resources awaiting incorporation there (Santos 2014; Lucas et al. 2016; Lima 2017; JBRJ 2018). For all analyses, we examined records present in 2007 (archived following earlier analyses) and in 2017. We converted locality coordinates to decimal degrees from the degree, minute, second values stored in the database.

### Classification of Changes to Database Records

To characterize how the database had changed, we classified differences between 2007 and 2017 records. At the highest level, these changes were additions (specimens not previously represented in the database) and changes in species name or locality coordinates or both (key fields).

We used a unique identification number (ID) to track records between 2 years. Additions were those with an ID not present in the first year. Changed records had an ID in both years, but the content of at least 1 key field had changed. Changes to the name field accounted for the largest proportion of changes between the years, so we further classified these to reflect their underlying drivers (Table 1).

**Table 1:** Classification of changes to specimen records in the *Myrcia* database

Major change	Minor change	Description	Short name	Number of changed records*
Additions		new specimens added to the database	new specimens	5663
Name change	nomenclatural	species name changed but circumscription did not	nomenclatural	2627
	taxonomic remodeling	specimens previously considered to represent a distinct species which was formally described now recognized as and included in a species described earlier	lumped	319
	taxonomic remodeling	subset of specimens from a species recognized as a distinct species not previously recognized in the database	split	111
	corrections to identifications	specimen misidentified and then corrected; represents either addition to the species that gains the specimen or subtraction from the species that no longer includes the specimen	correction in correction out	364
	rank	specimen originally identified to genus but since fully determined to species	upgraded to species	420
		specimen originally misidentified to species level, but level of certainty decreased and since identified only to genus	downgraded to genus	25
Geography	new coordinates	specimen previously lacking georeference has coordinates	new coordinates	225
	corrected coordinates	specimen with coordinates has new coordinates to improve accuracy or precision	corrected coordinates	78

^*^See Supporting Information for a breakdown of changes to records that altered extent of occurrence.

### Analysis of Changes to Database and Impacts

For a geographic overview of database differences between 2007 and 2017, we counted and mapped numbers of specimens and species per grid cell with an equal area projection and a grid‐cell size of 10,000 km.^2^

We generated preliminary IUCN assessments for each species from 2007 and then 2017 data, based on extent of occurrence (EOO), the metric most commonly used as the basis for IUCN Red List assessments for plants in the absence of population data (Brummitt et al. 2015). The EOO, the minimum area encompassing all known records, was calculated as a minimum convex polygon. We assigned a preliminary category of critically endangered (CR), endangered (EN), vulnerable (VU), near threatened (NT), or least concern (LC) to each species based on IUCN Red List Criterion B (IUCN 2012), but considering only the value of EOO (i.e., not in conjunction with subcriteria a–c). We treated species with <3 occurrences as data deficient (DD) because 3 is the minimum number of records required to calculate EOO. We calculated Sorensen's dissimilarity to compare turnover among species assigned to each category between 2007 and 2017 with the vegan package in R (Oksanen et al. 2017).

We carried out significance tests by Bayesian parameter estimation (Kruschke 2011). We estimated differences in the probability of a species having a particular conservation category and differences in specimens undergoing a particular change using multinomial likelihoods and estimated the difference in proportion of threatened species with a binomial likelihood. We chose the appropriate uninformative conjugate priors (Gelman et al. 2013) for ease of calculation and drew 10,000 samples directly from the modeled posteriors. We designated any difference as significant if 0 fell outside the 95% credible interval (CI) for the estimate.

For an overview of the changes to EOO, we plotted EOO 2007 against EOO 2017 for all species that appeared in both years. We applied 2017 identifications throughout to determine overall patterns of EOO changes in species. We evaluated relative impacts of different kinds of change to the database on EOO estimates for each species. For each individual specimen change, we calculated the impact on EOO for the corresponding species as follows. We used localities of all specimens for that species present in 2007, except for the single changed specimen for which the 2017 data was used. We calculated a hypothetical EOO based on this subset. For example, the hypothetical EOO involving a specimen with corrected coordinates was calculated using locality data from all 2007 specimens except for the changed specimen for which we used 2017 coordinates. We then subtracted this hypothetical EOO from the 2007 EOO to obtain a value for the increase or decrease in EOO attributable to that single specimen change. We excluded purely nomenclatural specimen changes from this analysis because; by definition, they did not change the locality data of a species and so could not change the EOO. For the small proportion of specimens that had undergone both taxonomic and geographic change, we attributed the hypothetical EOO change to the taxonomic change.

We carried out all analyses in R version 3.4.1 (R Core Team 2017) and used the circlize package (Gu et al. 2014) to generate chord diagrams and the rCAT package (Moat 2017) to calculate EOO and perform preliminary conservation assessments.

## Results

### Changes to the *Myrcia* Database

Between 2007 and 2017, there was a 50% increase in number of specimens represented in the database, in mean number of specimens per species, and in number of specimens with geolocation details (Supporting Information). Increased specimen numbers were primarily due to specimens collected before 2007 but databased later (79%) (Supporting Information). We identified and categorized 9817 specimen changes. The most frequent change was addition of new specimens (Table 1 & Supporting Information). No further changes were documented for these additions because they appear only in 2017. Of the 9727 specimen records present in the database in both years, 41% had undergone at least 1 change by 2017 and some underwent more than 1 type of change, purely nomenclatural changes were the most frequent (Table 1 & Supporting Information).

**Figure 1:**
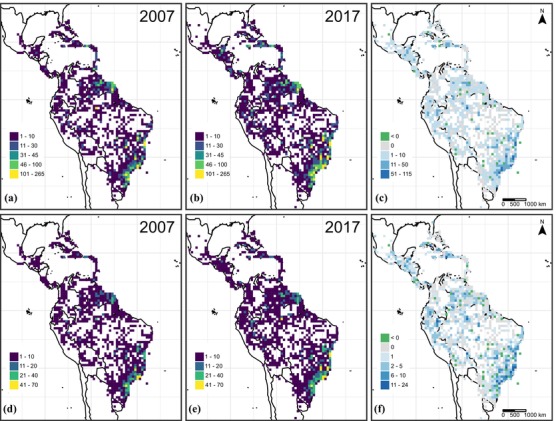
The number of *Myrcia* (a, b) specimens and (d, e) species represented in the database in 2007 and 2017 per 10,000‐km^2^ grid cell and the difference in (c) specimens and (f) species.

For historical reasons, most specimens in the database were from Brazil (Fig. 1a), from the Atlantic forest and Cerrado biomes and from French Guiana. After 2007, additions to the database significantly enhanced its geographic and taxonomic coverage such that 83% (667) of the c. 800 known species in the genus and over 50% of the c. 2000 grid cells within the range of the genus are now represented by at least 1 specimen. Areas of underrepresentation in the database include central and Amazonian Brazil, Colombia, Venezuela, Bolivia, and Peru (particularly from the Amazon). Between 2007 and 2017, collecting expeditions in the Brazilian Atlantic forests furthered specimen representation from there. Expeditions to Brazil's southern Amazon and donations of duplicate sets of Brazilian collections filled further gaps. Incorporation of specimens from collecting expeditions to the Caribbean, Ecuador, and Peru increased these regions’ representation. Patterns for species numbers are similar (Fig. 1b). The main differences were a slight increase in records from central Amazon and a more evident increase in Peru (Figs. 1c,d).

### Changes to Preliminary Conservation Assessments

**Figure 2:**
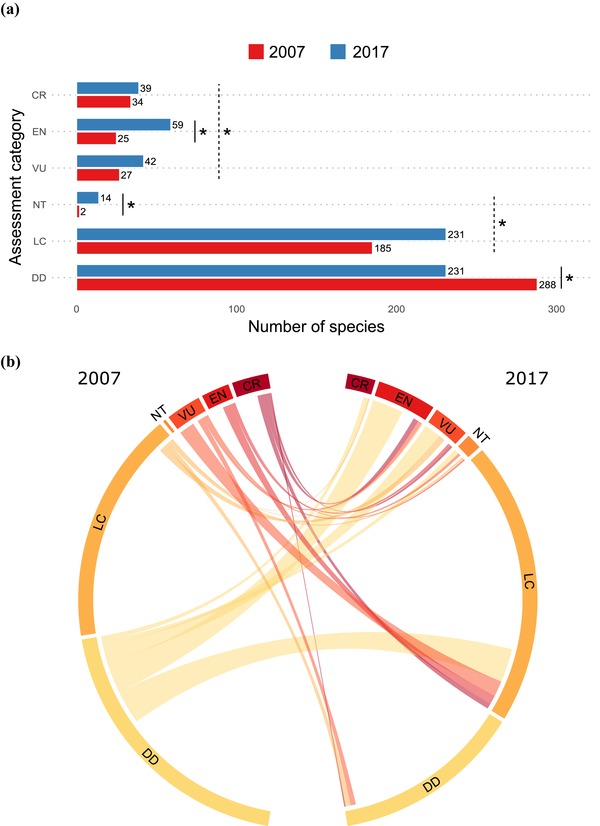
(a) The number of *Myrcia* species assigned to each conservation category by preliminary assessment based on calculations of extent of occurrence with locality data for each species from 2007 and 2017 (^*^, significant change) and (b) changes in preliminary assessment results based on locality data from 2007 and 2017 (CR, critically endangered; EN, endangered; VU, vulnerable; NT, near threatened; LC, least concern; DD, data deficient). Changes are significant if 0 is outside the 95% CI of the change (calculated by Bayesian parameter estimation).

Comparison of preliminary conservation assessments based on 2007 records with those based on 2017 records (Fig. 2a) showed that categories of most species were unchanged. However, there was a significant reduction in the number of species evaluated as DD and a significant increase in numbers of species evaluated as threatened (VU, EN, or CR) and in species evaluated as not threatened.

Most species evaluated as DD in 2007 but not in 2017 were evaluated as threatened or near threatened in 2017 (Fig. 2b). These formerly DD species accounted for most significant increases in species evaluated as NT and EN. Lack of significant change in number of VU or CR species (Fig. 2a) masked substantial species turnover in these categories. Turnover was highest for VU species (Sorensen dissimilarity = 0.84) (Supporting Information). Almost half of the species evaluated as VU in 2007 were treated as LC in 2017, but this reduction was more than offset by species formerly evaluated as DD transitioning to VU (Fig. 2b). Turnover was moderately high in CR species (Sorensen dissimilarity = 0.35) (Supporting Information). Among species categorized as CR in 2007, changes to EN or LC were equally common.

### Specimen Records Driving Change in Conservation Category

**Figure 3:**
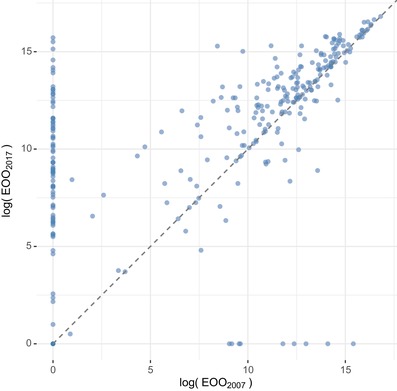
Comparison of the extent of occurrence (EOO) (km^2^) in 2007 and 2017 for all *Myrcia* species present in in the database in both years. Both axes are plotted on the log (*x*+1) scale.

The EOO estimates for most species (55%) were greater in 2017 than in 2007, but EOO was unchanged for many species and reduced for a substantial minority of species (11%) (Fig. 3). To explore drivers underlying these changes in EOO, we excluded purely nomenclatural changes that cannot influence conservation assessment, leaving 7190 specimen changes with potential to cause differences between EOO estimates in 2007 and 2017. Corrections to misidentifications accounted for 3.7% of changes to specimen records. Each correction to identification had the potential to alter the EOO estimate of 2 species. Where this was the case, they were counted as a correction out (i.e., a specimen subtracted from a species due to a corrected misidentification) or a correction in (i.e., a specimen added to a species due to a corrected misidentification) to create a subset of data focused on impacts on EOO. Like the dataset as a whole, this subset was dominated by new specimens, but corrections and refinements to existing identifications were also prominent (Supporting Information). About 10% of all specimen changes with potential to alter EOO affected species for which EOO changed sufficiently to alter the preliminary conservation category (Supporting Information). New specimens still dominated this subset, but coordinate changes and corrections or refinements to existing identifications were more prominent in this subset than in previous subsets.

**Figure 4:**
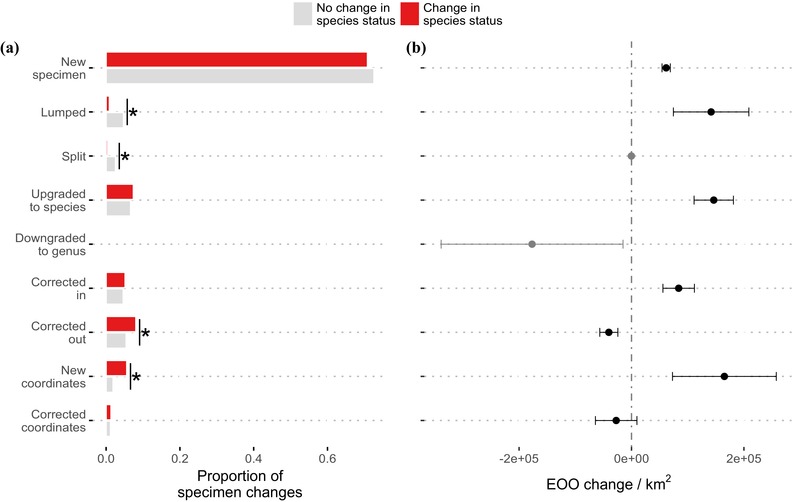
(a) Proportion of each type of specimen change associated with species that did and did not change preliminary conservation category between 2007 and 2017 (^*^, significant difference if 0 fell outside the 95% CI of the change [calculated by Bayesian parameter estimation]; corrected out, subtraction of a specimen from a species due to correction of misidentification; corrected in, addition of a specimen from a species due to correction of misidentification). (b) Mean change in extent of occurrence (EOO) for each type of specimen change (calculated as the change in EOO when only the single specimen change in question is in the locality data from 2007; whiskers, SE contribution for each specimen type).

Four types of specimen changes differed significantly in their representation in species that had and had not changed conservation category between 2007 and 2017 (Fig. 4a). Specimen changes resulting from 2 species being lumped together and changes resulting from 1 species being split from another each accounted for a significantly greater proportion of the total changes for species that did not change category than for species that did. In contrast, 2 types of specimen change were overrepresented among species that changed category between 2007 and 2017. New coordinates and corrections to existing identifications that resulted in 1 less specimen for the species of interest each accounted for significantly greater proportions of total changes in species that changed category than in species that did not.

Comparing mean impact on EOO of a single specimen change of each type, for most types of specimen change, the mean impact of a single specimen change was an increase in EOO estimate (Fig. 4b). Each new specimen added drove a relatively small but predictable increase in EOO. In contrast, we observed larger mean EOO increases from addition of coordinates to previously ungeoreferenced specimens, but these changes were more variable so their per‐specimen impacts did not differ significantly from those of new specimens. Mean impacts on EOO of specimens lumped, corrected in, or upgraded to species were all positive and intermediate in size and variability between those of new specimens and new coordinates. Corrections to identifications resulting in 1 less specimen record for the species of interest resulted, on average, in a small but significant reduction in EOO estimate, whereas corrections to coordinates had a similar mean effect but greater variance and did not differ significantly from 0.

## Discussion

Numbers of threatened and not threatened *Myrcia* species increased significantly over the study period, and there was a corresponding decrease in species deemed DD. Additions of specimen records and taxonomic remodeling had relatively little impact in driving changes in conservation category compared with corrections of misidentifications and enhanced georeferencing. Highlighting curatorial actions with greatest potential impact on conservation assessments can inform effective resource allocation to maximize return on investment in integrative monography and conservation science, but imperfect data and ongoing monography should not prevent conservation assessments.

### Advances in Taxonomy and Ecology of Neotropical Myrtaceae

Collections‐based taxonomy and systematics have underpinned extraordinary recent growth in understanding of Neotropical Myrtaceae diversity and its ecological and conservation significance. The parlous state of knowledge of Neotropical Myrtaceae in the late 20th century is portrayed in 2 1983 publications. A review of Southern Bahian Moist Forests highlights Myrtaceae as the most diverse and dominant family, but reports plot data where all 26 Myrtaceae species are identified only to family level (Mori et al. 1983b). Myrtaceae were found in almost all forest strata but no further ecological insights are offered, other than that sheer weight of numbers make them ecologically important (Mori et al. 1983a,1983b). Authors stress that ecological description of these exceptionally diverse forests awaits taxonomic revisions of Myrtaceae but that such revision might become impossible due to rapid disappearance of the forests.

From this low baseline, taxonomic knowledge of *Myrcia* developed steadily in the 1990s, and growth accelerated in the 21st century as study of Neotropical Myrtaceae entered the phylogenomic era. A key step was splitting *Myrcia* into smaller natural groups based on molecular evidence (Lucas et al. 2011). Subgeneric groups were formally published only when increased statistical and morphological support for the arrangement was obtained. In the interim, informal groups were adopted to guide studies and facilitate discussion. Splitting the megagenus into smaller workable units (Lucas et al. 2018) enabled coordinated research. The first phylogeny for the genus (Lucas et al. 2011) coincided with increased funding from Brazil, where *Myrcia* is most diverse (Govaerts et al. 2018). Successive grants allowed individual clades to be tackled cooperatively, yielding results including enhanced collections and taxonomic precision in the database.

Newly recognized sections enabled focused studies not only on taxonomy, but also on ecology, macroevolution, and distribution of diversity. For example, analysis of species’ relationships and macroevolutionary dynamics of *Myrcia* sect. *Aulomyrcia* in the Atlantic Forest showed lower extinction rates inside climatic refugia and high levels of lineage dispersal from unstable to stable areas, suggesting these processes maintained diversity in a region renowned for high biological diversity (Staggemeier et al. 2015). The sections also allow floristic studies to attribute more precise identifications than previously possible reliable species identification is the vital first step to ensure reproducibility in all biological studies and is particularly important in assessment of biodiversity for conservation. If species are not identified, one cannot evaluate extinction risk, prioritize, or protect species.

In contrast to earlier studies (Mori et al. 1983a,1983b), there is a growing understanding of the ecological importance of Neotropical Myrtaceae (e.g., in terms of their importance to forest fauna). The morphofunctional space of Myrtaceae fruits is particularly wide. These fruits sustain a range of animals (Staggemeier et al. 2017) with varied energetic requirements, such as birds (e.g., tanagers), frugivores (e.g., guans and toucans), and mammals (e.g., rodents, tapirs, monkeys). Furthermore, although seasonal flowering patterns are concentrated in months with warmer and longer days, Neotropical Myrtaceae offer fruits throughout the year, likely due to differences in seed physiology (Staggemeier et al. 2010, 2017). Such insights serve to strengthen the case for in situ conservation of Myrtaceae species as key sources of food for charismatic animal species.

### Stabilizing Taxonomy for Conservation

Although our study was designed before recent calls for stronger governance to stabilize taxonomy in the interests of conservation (Garnett & Christidis 2017), our results inform the debate. Changes to species data resulting from taxonomic remodeling (lumping or splitting) showed a quite different pattern from changes due to correcting misidentifications. Changes from lumping or splitting were significantly underrepresented among species that changed preliminary conservation category, suggesting that taxonomic remodeling is not a key driver of changes to conservation category in *Myrcia*, a finding likely applicable to other large, long‐overlooked tropical plant genera. We contend that results from *Myrcia*, a large group in which species outnumber specialists by 100 to 1, are likely more representative of patterns to be expected in other large, understudied plant clades (e.g., *Croton, Eugenia, Miconia, Ocotea, Psychotria, Syzygium*) and understudied insect groups than well‐known vertebrate groups. Rather than additional time‐consuming governance for taxonomy, the interests of conservation are best served by more collaboration among taxonomists, ecologists, and conservation scientists (Dayrat 2005; Baker et al. 2017) to enhance translation of taxonomic insights into ecological understanding and management of species on the ground.

Frequency of plant misidentifications and their impact on results and conclusions are particularly intractable questions (Meyer et al. 2016). At 3.7%, the proportion of misidentifications detected and corrected in our database over the decade is toward the low end of published values (Meyer et al. 2016) but higher than other studies of tropical trees. Misidentification rates <2% are reported for the entire tree flora of Barro Colorado Island (Condit 1998), for Tanzanian trees (Ahrends et al. 2011), and for *Inga* in Amazonian Peru (Dexter et al. 2010), although misidentification rates >30% are reported for difficult genera in western Amazonia, including *Inga* (Baker et al. 2017). In our study, corrected misidentifications were overrepresented among species, which changed preliminary conservation category. These results highlight the importance of ongoing conscientious database curation, capturing changes to specimen identifications as they are made.

The database cannot ever precisely reflect the natural world but remains the most comprehensive, continuously curated resource for *Myrcia*. Although over 80% of specimens are georeferenced, achieving complete georeferencing remains unlikely since specimens lacking coordinates include historic collections with imprecise locality details. Our analyses highlight the significance of each newly georeferenced specimen to refining extinction risk estimates and reinforce the need for further, focused georeferencing activity.

Although conservation zoologists have tracked accumulation of knowledge about species over time, often focusing on distinguishing change in knowledge from genuine change in species’ status (Hoffmann et al. 2010), plants have received relatively little attention of this kind. In a study of Madagascar orchids, Roberts et al. (2016) found that most species’ area‐accumulation sequences differ significantly from random; early samples show a greater proportion of species’ known EOO than predicted from random processes. Their conclusions lend support for assessing species on best currently available evidence rather than delaying assessments of extinction risk until area‐accumulation curves flatten.

Extinction risk assessments underpin area prioritization for conservation (Darbyshire et al. 2017). The *Myrcia* database was a key resource in recent consultations on criteria and thresholds for recognition of Key Biodiversity Areas (IUCN 2016). Since mid‐2017, the *Myrcia* database has supported a new initiative, to complete extinction risk assessments for all *Myrcia* species by 2021. Now is the right time for this ambitious endeavor. Collectively, the changes documented increased the number of species represented in the database and numbers of specimens documented per species, driving significant decreases in numbers of species categorized as DD and greatly reducing uncertainty regarding the conservation status of *Myrcia* as a whole. Without extinction‐risk assessments, *Myrcia* cannot be fully factored into in situ conservation plans—a grim prospect for a genus with recalcitrant seeds not amenable to conventional seed‐banking approaches. Prospective assessors should not be deterred by gaps in data or the group's taxonomic complexity. Within IUCN Red List guidelines even poorly known and complex groups can be assessed, as various levels of data uncertainty can be factored in, provided they are justified and evidence based. Over 100 *Myrcia* species have yet to be researched and included in the *Myrcia* database. Inevitably, more species will be evaluated as DD, and some category assignments will change as new data become available in the course of the project. Outcomes of analyses such as ours may change with improved data and analytical techniques. Nevertheless, if species are not assessed and factored into conservation actions soon, the potential for evolutionary and ecological studies will be lost along with the species’ environments.

### Future Directions

Our results highlight challenges and opportunities for diverse stakeholders. For curators of herbaria and digital catalogues in which data are disseminated, the nature and scale of changes to our relatively small data set may be no surprise, but their significant impact on conservation assessments will interest curators advocating use of collections for conservation biology. Enabling access to a digital representation of the collections is just the first step in this regard. Unless digital versions are updated as physical collections change (and vice versa, if appropriate), utility is rapidly eroded. But cash‐strapped herbarium curators are already finding that, once externally funded digitization projects end, the time required in maintaining the digital catalogue as a faithful representation of the physical collection is unaffordable (S. Phillips, personal communication).

Georeferencing, key to informing conservation, is also unaffordable from most curation budgets and usually undertaken as discrete projects where subsets of data are downloaded and georeferenced. Value added to these subsets may never reach the source database, even where the will exists, because technical routes to achieve it are lacking. Thus, some collections are independently georeferenced repeatedly, from the same specimen record or from duplicates deposited in different herbaria, with varying levels of accuracy and precision, while others are overlooked. Greater collaboration and integration of georeferencing efforts is arguably the most significant opportunity for herbarium collections to enhance their conservation value. Large‐scale, accurate georeferencing of specimens by trained individuals should be budgeted into funding proposals for digitization and research.

For monographers contemplating revisions of large, intractable clades, collaboration is a key (Knapp 2008). Teamwork by researchers brings different taxonomic, technical, and geographical perspectives, greatly enriching specimen databases and offering vast potential for ecological and conservation‐based study. Monographers wishing to maximize conservation relevance of their work must ensure that taxonomic changes to specimens are promptly incorporated in specimen databases and available for analysis.

Our dissection of a large genus, the herbarium specimens through which it is studied, their changing digital representations and resulting conservation inferences, highlights important considerations for conservation science. Robust, evidence‐based taxonomy is critical in making NHCs useful for conservation because it allows authoritative identification of units of evolution. Conservation scientists relying on digital specimen records should ensure that these reflect current taxonomic and geographic knowledge and not merely a snapshot of NHC data when they were first digitized. Where resource constraints limit updates, correction of misidentifications and georeferencing of existing records should be prioritized in light of their significant impacts on conservation assessment. Groups lacking conservation assessments cannot easily be factored into conservation prioritization and planning, but assessments based on poorly circumscribed entities lack credibility. Attempts to artificially stabilize taxonomy cannot solve this problem and would be counterproductive for both systematics and conservation. Instead, for large, complex groups, we recommend an integrative monography approach in which conservation analyses accompany taxonomic revision, rather than a sequential process whereby conservation inferences are deferred until taxonomy is finalized.

## Supporting information

Data analyzed (Appendix S1), collection years of post‐2007 additions to the database (Appendix S2), proportions of specimen changes 2007–2017 (Appendix S3); Sorensen dissimilarities (Appendix S4), mean EOO changes for specimen change types (Appendix S5), and statistical overview of *Myrcia* database (Appendix S6) are available online. The authors are solely responsible for the content and functionality of these materials. Queries (other than absence of the material) should be directed to the corresponding author.
