# Supplementary material for: Harnessing the potential of integrated systematics for conservation of taxonomically complex, megadiverse plant groups
Source: Conserv Biol. 2019 Feb 19;33(3):511–22. doi: 10.1111/cobi.13289 (PMC6850456; doi:10.1111/cobi.13289)
Supplement: Supplementary file 1 — Data analyzed (Appendix S1), collection years of post‐2007 additions to the database (Appendix S2), proportions of specimen changes 2007–2017 (Appendix S3); Sorensen dissimilarities (Appendix S4), mean EOO changes for specimen change types (Appendix S5), and statistical overview of Myrcia database (Appendix S6) are available online. The authors are solely responsible for the content and functionality of these materials. Queries (other than absence of the material) should be directed to the corresponding author. [file COBI-33-511-s001.docx]

**Supporting Information**

**Article: Harnessing the potential of integrated systematics for the conservation of taxonomically complex, megadiverse plant groups**

**Appendix S1**

Data used to analyze the impact of changes to specimen records held in a monographic database are provided on Figshare (https://doi.org/10.6084/m9.figshare.6148313). These comprise a table containing the species, coordinates, determination level, and record id of each specimen record in the two snapshots of the database, and a table of classified name changes that occurred between the two snapshots. All species names have been given a unique identifier due to data sensitivity concerns relating to threatened species and data supplied by partners.

**Appendix S2**


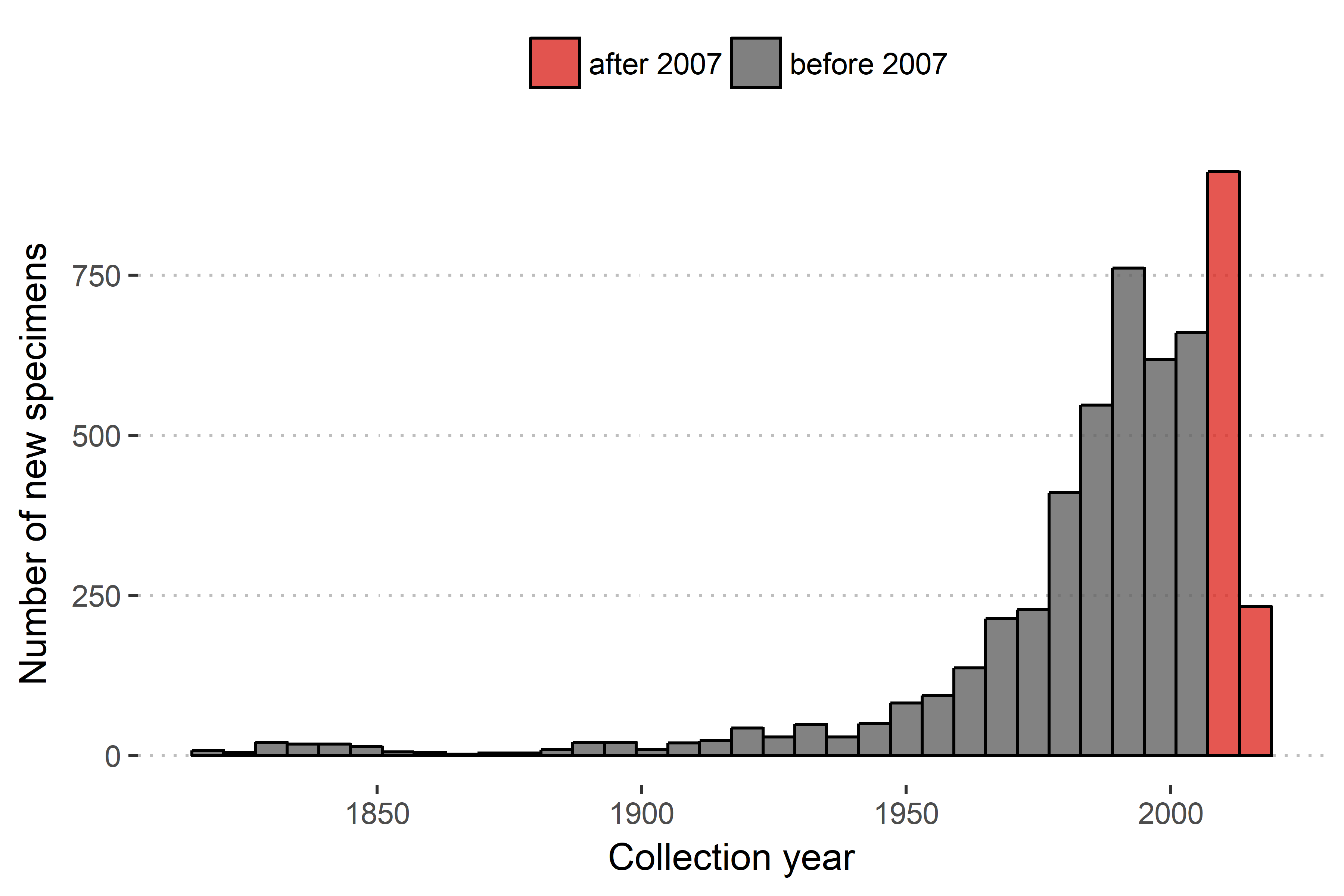
 Histogram of the collection year of all specimens new to the database in the 2017 snapshot, contrasting those that were new to the database but collected before 2007 with those that were newly collected after the 2007 snapshot.

**Appendix S3**

**
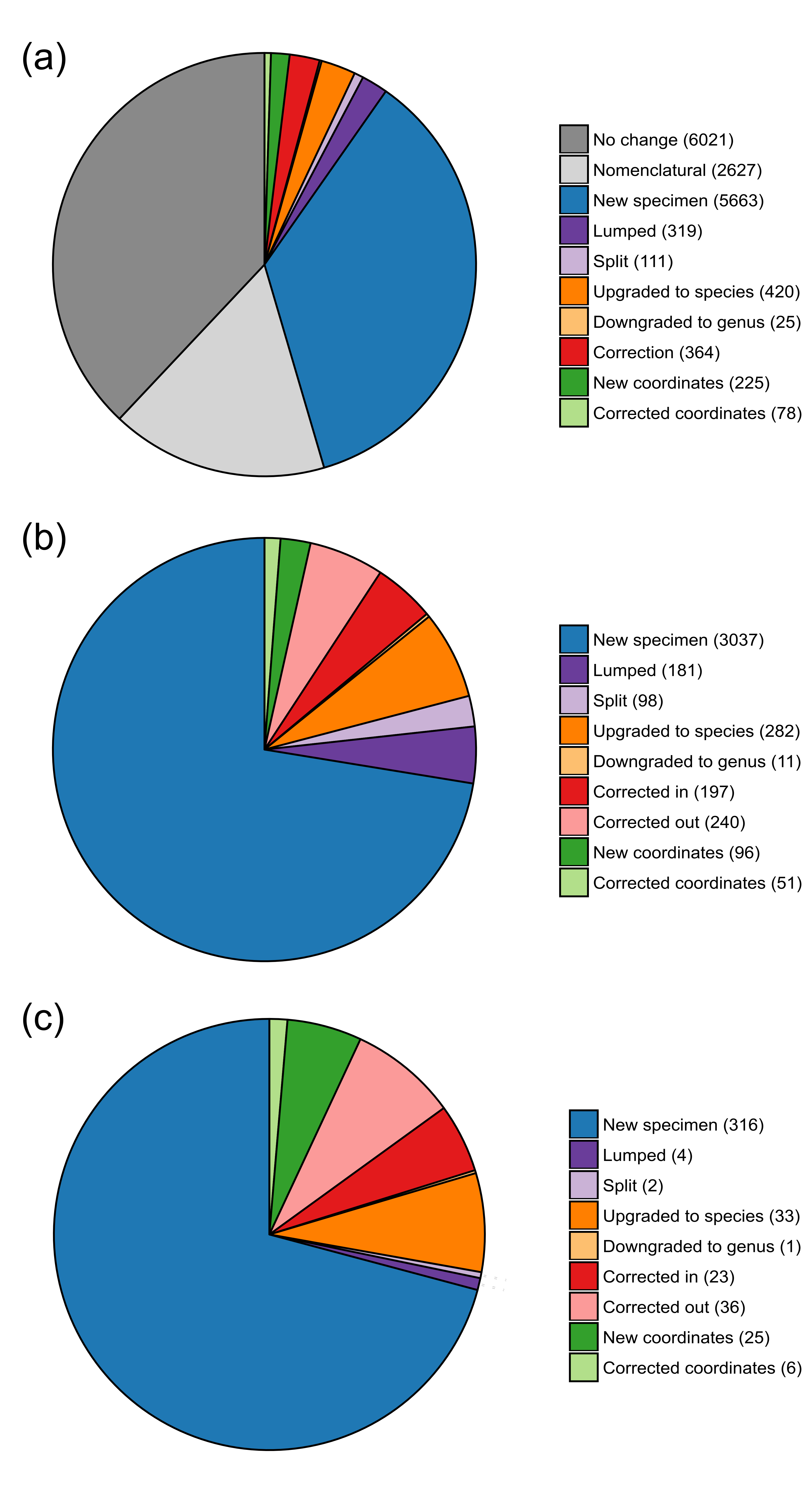
**

Proportions of *Myrcia* specimen changes between 2007-2017 by type for (a) all specimen changes, (b) only specimen changes that altered EOO, and (c) only specimen changes that altered EOO and were associated with species that did change extinction risk category. Actual numbers are reported in parentheses.

**Appendix S4**

**
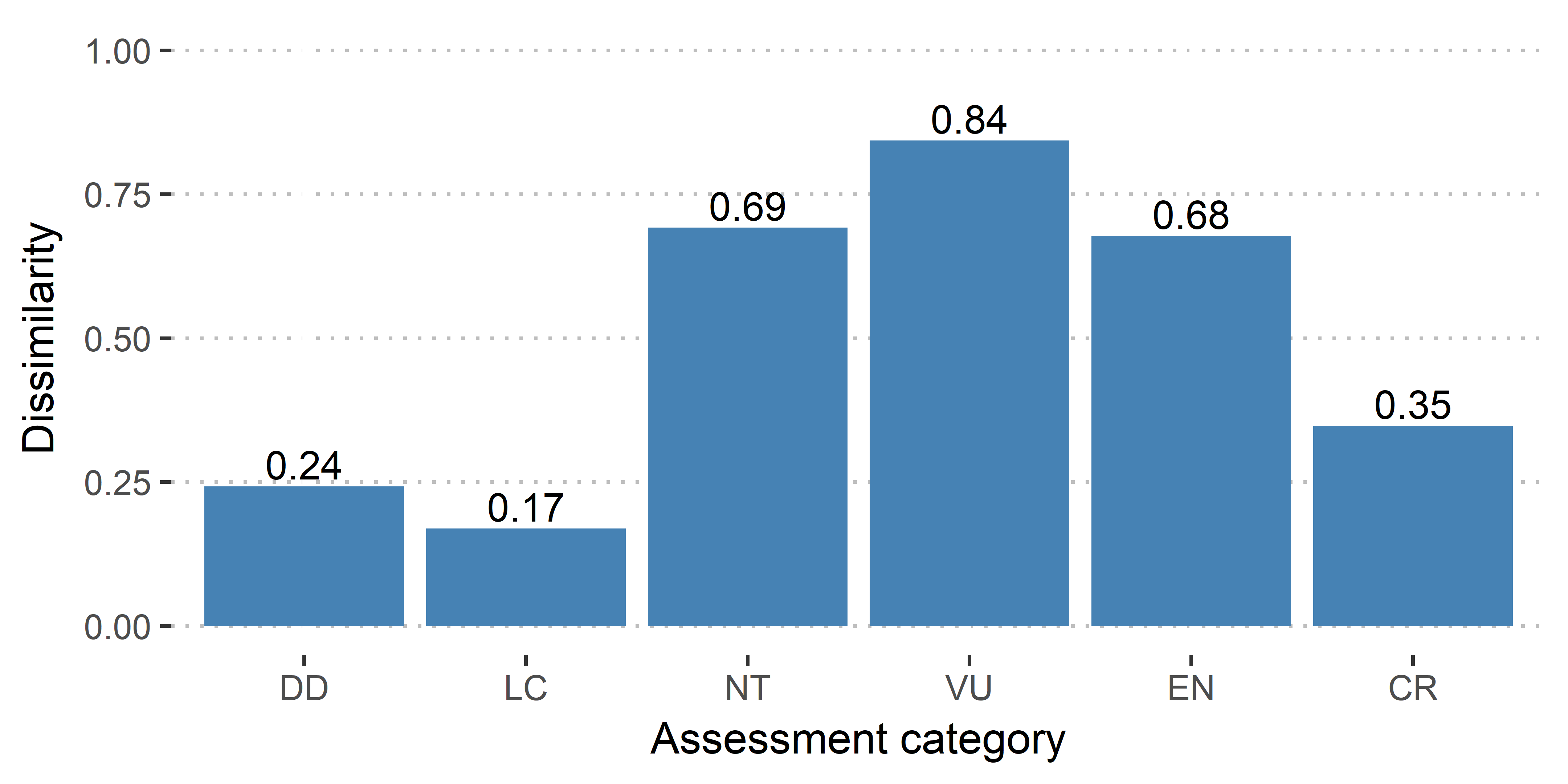
**

Sorensen dissimilarity between species with each preliminary conservation assessment category in the 2007 and 2017 database snapshots. Preliminary categories: critically endangered (CR), endangered (EN), vulnerable (VU), near threatened (NT), least concern (LC), and data deficient (DD).

**Appendix S5**


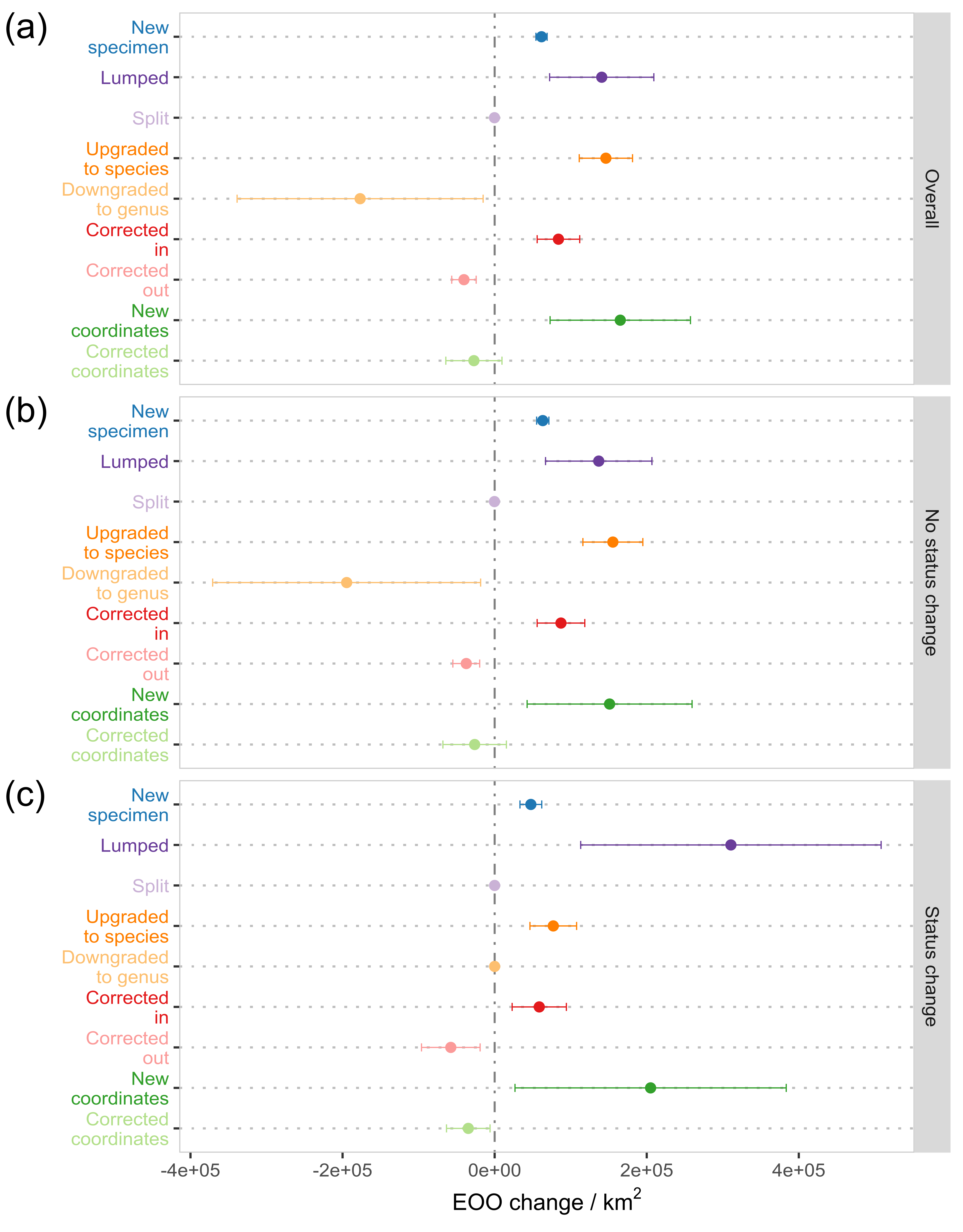


The mean change in EOO for each specimen change type, calculated as the change in EOO when only the individual specimen change in question is incorporated into the locality data from 2007, for (a) all species, (b) only species that did not change extinction risk category, and (c) only species that did change extinction risk category. Error bars indicate the standard error of the mean contribution for each specimen change type.

**Appendix S6**

| A summary of the data held in the *Myrcia* database in 2007 and 2017. | | |
| --- | --- | --- |
|  |  |  |
|  |  |  |
| **Metric** | **2007** | **2017** |
| Number of grid cells covered (cell size: 10,000 km^2^) | 872 | 1040 |
| Number of species in the database | 643 | 667 |
| Species per cell (mean) | 4.5 | 5.3 |
| Species per cell (mean, 2007 cells*) | 4.5 | 6.0 |
| Species per cell (median) | 2 | 3 |
| Species per cell (median, 2007 cells*) | 2 | 3 |
| Specimens | 10111 | 15390 |
| Specimens identified only to genus | 1201 | 998 |
| Specimens per cell (mean) | 9.3 | 5.3 |
| Specimens per cell (mean, 2007 cells*) | 9.4 | 13.8 |
| Specimens per cell (median) | 3 | 3 |
| Specimens per cell (median, 2007 cells*) | 3 | 4 |
| Specimens per species (mean) | 13.9 | 21.6 |
| Specimens per species (median) | 2 | 5 |
| Specimens with geolocation | 8125 | 12350 |
|  |  |  |

* Values reported for ‘2007 cells’, refer to mean or median calculated across cells which had records for at least one specimen and species in 2007.
